# AMP-Activated Kinase Regulates Lipid Droplet Localization and Stability of Adipose Triglyceride Lipase in *C*. *elegans* Dauer Larvae

**DOI:** 10.1371/journal.pone.0130480

**Published:** 2015-06-22

**Authors:** Meng Xie, Richard Roy

**Affiliations:** Department of Biology, McGill University, 1205 avenue Docteur Penfield, Montreal, Canada; CSIR-Central Drug Research Institute, INDIA

## Abstract

Animals have developed diverse mechanisms to adapt to their changing environment. Like many organisms the free-living nematode *C*. *elegans* can alternate between a reproductive mode or a diapause-like "dauer" stage during larval development to circumvent harsh environmental conditions. The master metabolic regulator AMP-activated protein kinase (AMPK) is critical for survival during the dauer stage, where it phosphorylates adipose triglyceride lipase (ATGL-1) at multiple sites to block lipid hydrolysis and ultimately protect the cellular triglyceride-based energy depot from rapid depletion. However, how the AMPK-mediated phosphorylation affects the function of ATGL-1 has not been characterised at the molecular level. Here we show that AMPK phosphorylation leads to the generation of 14-3-3 binding sites on ATGL-1, which are recognized by the *C*. *elegans* 14-3-3 protein orthologue PAR-5. Physical interaction of ATGL-1 with PAR-5 results in sequestration of ATGL-1 away from the lipid droplets and eventual proteasome-mediated degradation. In addition, we also show that the major AMPK phosphorylation site on ATGL-1, Ser 303, is required for both modification of its lipid droplet localization and its degradation. Our data provide mechanistic insight as to how AMPK functions to enhance survival through its ability to protect the accumulated triglyceride deposits from rapid hydrolysis to preserve the energy stores during periods of extended environmental duress.

## Introduction

Most organisms have little to no control over their environment and therefore need to adjust their behaviour and physiology accordingly in response to the challenges posed by their surroundings. In hibernating mammals, environmental cues trigger significant changes in foraging behaviour and metabolism to enhance survival during winter [1]. Other organisms use a reproductive trade off to enhance survival: stress, be it either physical or energy stress, can cause hormonal imbalance and reproductive arrest to divert limiting macromolecules for survival needs rather than reproduction [2, 3, 4].

Like many organisms, the free-living nematode *C*. *elegans* can divert reproductive development and execute an alternative developmental pathway referred to as the "dauer" stage. This modified third larval stage provides the animals with increased stress resistance that enables them to circumvent suboptimal growth conditions such as nutrient deprivation, high temperatures, or elevated population density. This highly resistant dauer stage is associated with reproductive arrest and allows animals to survive several months in this juvenile state, compared to the normal adult lifespan of 14 days [5, 6]. One unique feature of the dauer larva is that the animals stop feeding upon entering the dauer stage, where they rely solely on their internal energy stores mainly in the form of triglycerides that accumulate in their intestinal and hypodermal cells prior to dauer formation [6].

The accumulated triglycerides are stored in lipid droplets, which are monolayer phospholipid-encapsulated organelles, the triglyceride core of which can be accessed in a regulated manner according to metabolic need. To mobilize these energy-rich molecules, a series of lipase-specific sequential reactions must occur to produce three free fatty acid molecules (one from each reaction) and glycerol, from each triglyceride molecule. The initial rate-limiting step of this lipolysis process is catalyzed by the enzyme adipose triglyceride lipase (ATGL) to release a single free fatty acid from a triacylglycerol substrate. *ATGL* disruption in mice has been shown to lead to triglyceride accumulation within multiple tissues [7].

In *C*. *elegans*, the "all or none" dauer entry response is determined by parallel cross-talk among several genetic pathways, including the insulin-like signaling pathway. AMP-activated protein kinase (AMPK) is a common target of these pathways, where the appropriate storage of lipid, osmoregulatory homeostasis, and germline stem cell quiescence are largely dependent on its action during dauer progression [8, 9].

AMPK exists as a heterotrimeric complex composed of a catalytic (α) subunit and two regulatory (β and γ) subunits in all organisms. Unlike in many other organisms where disruptions of AMPK function are lethal, AMPK null mutants are viable under normal growth conditions in *C*. *elegans*. However, in insulin signaling-compromised dauer animals that lack both AMPK catalytic subunit isoforms (encoded by *aak-1* and *aak-2*), the germline stem cells proliferate extensively causing a germline hyperplasia phenotype [8], while the animals expire prematurely due to rapid exhaustion of their lipid reserves [9].

Curiously, although hormone sensitive lipase (HSL) has been shown to be a direct phosphorylation target of AMPK in rat muscle cells [10], loss of HSL does not affect the rapid depletion of lipid stores observed in *C*. *elegans* dauer larvae [9]. Alternatively, the *C*. *elegans* homologue of ATGL (ATGL-1) was found to be a direct phosphorylation target of AMPK at multiple residues, where Serine 303 is the predominant phosphoacceptor residue [9]. Phosphorylation of ATGL-1 by AMPK during the dauer stage limits lipolysis, allowing the establishment of a triglyceride depot that provides the essential energy for the long-term survival of the non-feeding nutrient-deprived dauer larvae.

The manner in which AMPK acts upon ATGL-1 to protect the triglyceride stores during the dauer stage could not be determined from our initial study. In one scenario AMPK could allosterically affect ATGL-1 activity through phosphorylation-dependent conformational change that affects the efficiency of the catalytic core (i.e. reducing its ability to bind its substrates). Alternatively, phosphorylation could affect changes to ATGL-1 stability or localization, blocking its access to the triglyceride substrate encapsulated in the lipid droplet. To probe the regulatory inputs that control this rate-limiting step in triglyceride breakdown we generated an antibody specifically against the *C*. *elegans* ATGL-1 protein. Our analyses revealed that phosphorylation of ATGL-1 resulted in a change in its subcellular localization away from the lipid droplets followed by its proteasome-dependent degradation, involving ubiquitylation and its prior interaction with a 14-3-3 protein; where the AMPK-mediated phosphorylation of Serine 303 residue is critical for both processes.

## Materials and Methods

### Strains and Reagents

*C*. *elegans* strains were cultured as previously described by Brenner [11]. Strain VS20 *hjIs67[Patgl-1*::*atgl-1*::*GFP]* [12] was obtained from CGC and subsequently crossed into CB1370 *daf-2(e1370)* and MR1000 *daf-2(e1370); aak-1(tm1944); aak-2(ok524)* strains. Strains MR1348 *daf-2(e1370); rrEx226[sur-5p*::*GFP*::*ATGL-1*::*HA; rol-6(gf)]* and MR1413 *daf-2(e1370); rrEx239[syr-5p*::*GFP*::*ATGL-1(S303A)*::*HA; rol-6(gf)]* are described in [9]. The strain *atgl-1(tm3116)* harbors a 423bp deletion of the *atgl-1* gene and was obtained from National BioResource Project, Tokyo, Japan. Rabbit polyclonal antibody against ATGL-1 was raised using a synthetic peptide CTKRKVPDEPTTSKR (GenScript). Anti-PAR-5 antibody was a gift from Dr. Andy Golden. Anti-GFP (abcam ab290), anti-ubiquitin (Santa Cruz SC8017) and anti-P-14-3-3 (Cell Signaling #2981S) antibodies are available commercially.

### Feeding RNAi

Our RNAi feeding protocol was performed as previously described [13]. Briefly, synchronized L1 animals were added onto regular plates seeded with individual dsRNA-expressing bacterial clones and maintained at 15°C. Phenotypes were scored thereafter.

### Immunoprecipitation and Western Blotting

*C*. *elegans* larvae and adults were lysed by sonication in lysis buffer (50mM Hepes pH7.5, 150mM NaCl, 10% glycerol, 1% Triton X-100, 1.5mM MgCl_2_, 1mM EDTA and protease inhibitors) and then incubated with anti-ATGL-1 or anti-PAR-5 antibody. Immunoprecipitations were performed with Protein-A agarose followed by immunoblotting with anti-ubiquitin, anti-P-14-3-3, anti-PAR-5 or anti-ATGL-1 antibody. Protein concentration was determined using a NanoDrop 2000c spectrophotometer (Thermo Scientific).

### Lipid Droplet Staining by C1-BODIPY-C12, Imaging, Volume and Quantity Quantification

C1-BODIPY-C12 staining was performed as described [14]. Briefly, synchronized L1 larvae were transferred to regular plates with C1-BODIPY-C12 and grown at 25°C. Images were acquired on a LSM510 confocal microscope (Zeiss) using a x40 1.3 oil objective. Lipid droplet diameter was measured using AxioVision (Zeiss) software and volume was calculated using the following formula: 4/3 x π x (diameter/2)^3^. Total lipid droplets per animal were quantified manually.

### Lipid Droplet Isolation

Lipid droplet isolation was performed as described with slight modification [15]. Briefly, animals fed with OP50/OP50+C1-BODIPY-C12 were washed with 1xPBS + 0.001% Triton X-100 and subsequently collected in Buffer A (25mM Tris pH7.6, 25mM glycine, 120mM sucrose and protease inhibitors) and kept on ice for 15min. The cells were lysed by adding liquid nitrogen and ground using a pre-chilled metal homogenizer with a tight-fitting pestle for 20 strokes on ice. The homogenates were centrifuged at 1000g for 10 min at 4°C to remove cell debris. The supernatant was then collected and centrifuged at 10,0000g for 1 hour at 4°C. The top white layer containing the lipid droplets was collected, resuspended with Buffer A and centrifuged 10,0000g again for 1 hour at 4°C to avoid cytosol and other membrane compartment contamination. Isolated lipid droplets were verified by C_1_-BODIPY-C_12_ staining, protein expression pattern and triglyceride enrichment compared to the supernatant after the first spin at 10,0000g before any further analysis.

### RNA Isolation, Real Time PCR and Semi-quantitative RT-PCR

Total RNA was extracted using Trizol (Invitrogen) as described [16]. The extracted RNA was purified by using an RNeasy Mini kit (Qiagen). The RNA concentration was determined by using a NanoDrop 2000c spectrophotometer (Thermo Scientific). The purity of the RNA was determined by measuring the absorbance at 260/280 nm. An OD_260/280_ ratio between 1.8 and 2 was considered sufficiently high enough quality RNA for RT-PCR. 0.5μg of purified RNA was used to synthesize cDNA. Gene expression levels were determined by real time PCR using the SYBR Green Supermix and BioRad iCycler Real Time PCRSystem (BioRad). Relative gene expression was normalized to *act-1* (gtcggtatgggacagaagga; gcttcagtgaggaggactgg) and *cdc-42* (tggcgagccatacacattag; ctttgagcaatgatgcgaaa) as internal loading control. Semi-quantitative RT-PCR reactions were performed with 150ng RNA using *GFP* and *act-1* (ggttgccgctcttgttgtag; ttagaagcacttgcggtgaa) specific primers.

### Ethics Statement

NA

## Results

### ATGL-1-Deficient Dauer Larvae Have Abnormally Large Lipid Droplets

The progressive accumulation of triglycerides during onset of dauer development presumably takes place to provide an energy stockpile that will sustain the larva during the course of a long diapause period [17]. To better understand how this stockpile is accrued and protected during this period of quiescence we genetically induced dauer formation and examined the dynamics of lipid droplets, the organelles that regulate lipid storage and utilization in most animals [18]. The dauer formation-2 (*daf-2*) gene encodes an insulin-like receptor in *C*. *elegans*, loss of function mutation of which leads to constitutive dauer entry at restrictive temperature (25°C) and is used as the control reference background for dauer entry throughout our work.

**Fig 1:**
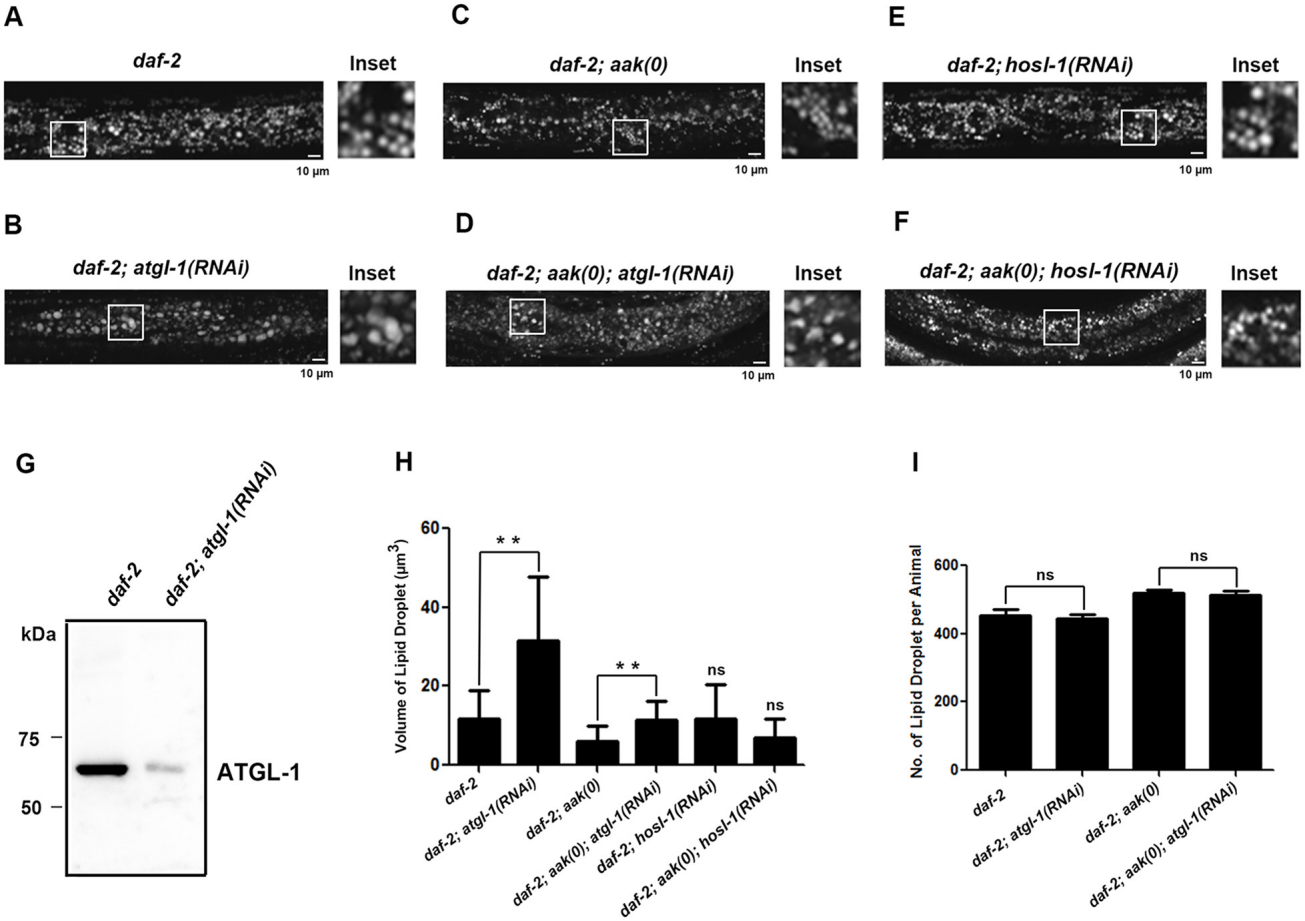
ATGL-1 Compromise Causes Enlarged Lipid Droplets in *C*. *elegans* Dauer Larvae. **(A)-(F)** Disruption of ATGL-1, but not HSL function in both control *daf-2* **(A)**, **(C)** and **(E)** and *daf-2; aak(0)* **(B)**, **(D)** and **(F)** dauer day 0 animals (48 hours after shifting to restrictive temperature since L1 stage) caused increase in lipid droplet size. Animals were stained with C_1_-BODIPY-C_12_. These and all subsequent images were taken with a Zeiss 510 Meta Confocal Laser Microscope at x40 magnification using identical microscope settings, unless specified otherwise. Scale bar = 10μm. Insets were generated by selecting the same size of frame on each image and amplified to the same magnification. **(G)** *atgl-1*(RNAi) feeding reduces endogenous ATGL-1 protein substantially. A Western blot probed with an anti-ATGL-1 polyclonal antibody raised against endogenous ATGL-1 was used to quantify ATGL-1 levels in control *daf-2* animals and *daf-2* animals subjected to feeding *atgl-1(RNAi)*. **(H)-(I)** Quantification of the volume **(H)** and number **(I)** of C_1_-BODIPY-C_12_-stained lipid droplets using AxioVision (Zeiss) software. ** indicates statistical significance (P<0.0001) and ns indicates not significant using the unpaired t test compared to control *daf-2* and *daf-2; aak(0)* animals respectively. Error bars indicate SD of three independent experiments.

Given that ATGL-1-deficient dauer larvae were shown to have more triglycerides [9], we first questioned whether the excess triglyceride molecules might have an effect on the lipid droplet size and/or number. Therefore, we stained dauer day 0 (defined as 48 hours after shifting to restrictive temperature at the L1 stage) control *daf-2* and *daf-2; aak-1; aak-2* (*aak-1; aak-2* will be presented as *aak(0)* hereafter) dauer larvae that were previously fed with regular or *atgl-1*(RNAi) bacteria using C_1_-BODIPY-C_12_ to label their lipid droplets (Fig 1A–1D). The efficiency of our feeding *atgl-1*(RNAi) protocol was confirmed with a *C*. *elegans* ATGL-1-specific antibody (see description below; Fig 1G). We found that following the compromise of *atgl-1*, the lipid droplet size was significantly increased in both control *daf-2* and *daf-2; aak(0)* dauers (Fig 1H) with no significant changes in lipid droplet number (Fig 1I). This indicates that triglycerides accumulate during the dauer entry phase but because they are not hydrolyzed due to the absence of ATGL-1 they remain encapsulated in the lipid droplets, leading to the enlargement of the organelles. Notably, the removal of hormone sensitive lipase (*hosl-1*), did not significantly alter the lipid droplet abundance or structure in either control *daf-2* or *daf-2; aak(0)* animals (Fig 1E, 1F and 1H), consistent with its secondary role in lipid hydrolysis during dauer survival [9].

### AMPK Regulates ATGL-1 Abundance during Dauer Entry and the Early Dauer Stage

**Fig 2:**
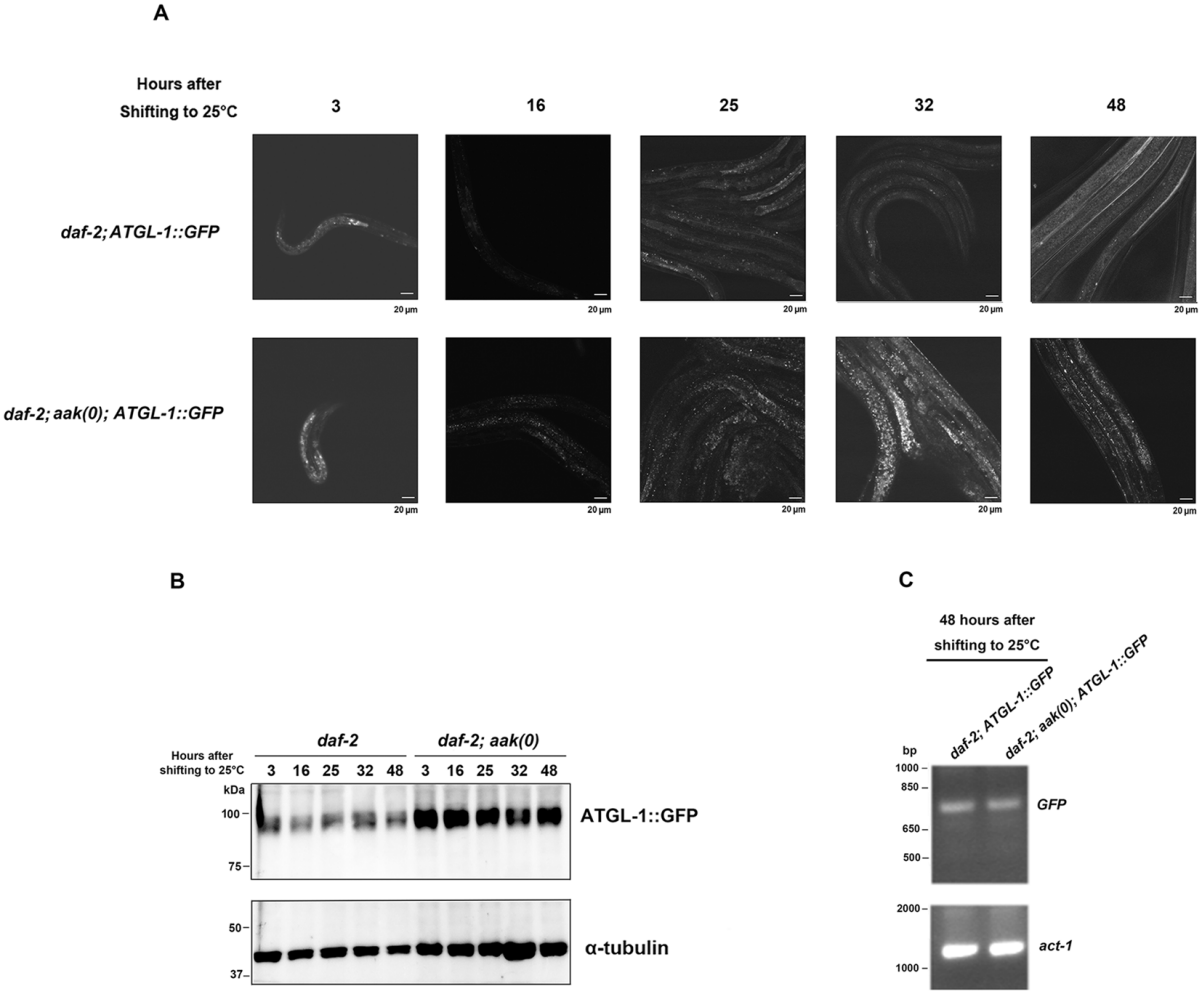
ATGL-1::GFP Accumulates to Higher Levels in AMPK-deficient Animals Prior to Dauer Entry. **(A)** Comparison of ATGL-1::GFP levels between control *daf-2* and *daf-2; aak(0)* animals during the dauer entry period. Images were taken with a Zeiss 510 Meta Confocal Laser Microscope at x20 magnification. All strains harbor the same *hjIs67[Patgl-1*::*atgl-1*::*GFP]* transgenic array in **(A)** and **(B)**. Scale bar = 20μm. **(B)** Western blot analysis of ATGL-1::GFP levels as measured using an anti-GFP antibody in control *daf-2* and *daf-2; aak(0)* mutant animals during the period prior to dauer entry. **(C)** Quantification of ATGL-1::GFP mRNA levels in control *daf-2* and *daf-2; aak(0)* mutant dauer day 0 animals using semi-quantitative RT-PCR. *act-1* was used as loading control.

To discern whether AMPK regulates ATGL-1 during the dauer stage through a possible allosteric effect of the phosphorylation versus an effect on ATGL-1 stability or localization, we monitored the fate of ATGL-1 after being phosphorylated by AMPK during this stage. Because AMPK phosphorylation can often trigger proteasome-mediated degradation [19], we wondered whether this possibility could explain the AMPK-mediated reduction in ATGL-1 function during the dauer stage. We therefore introduced a fully functional ATGL-1::GFP translational fusion protein into control *daf-2* and *daf-2; aak(0)* dauer larvae to compare ATGL-1 expression levels in these animals. We documented the ATGL-1::GFP expression in these animals at various time points during the entire dauer entry period, which we define here as the first 48 hours after being shifted to restrictive temperature (Fig 2A). ATGL-1::GFP was significantly more abundant in the absence of AMPK at all the time points during the entire dauer entry period (Fig 2B).

In addition, to determine whether any changes we observed occurred at the level of gene expression we assessed the mRNA levels of ATGL-1::GFP in control *daf-2* and *daf-2; aak(0)* dauer day 0 animals using GFP specific primers and observed no difference between the two, indicating that ATGL-1 is regulated post-transcriptionally by AMPK most probably by affecting protein stability (Fig 2C).

**Fig 3:**
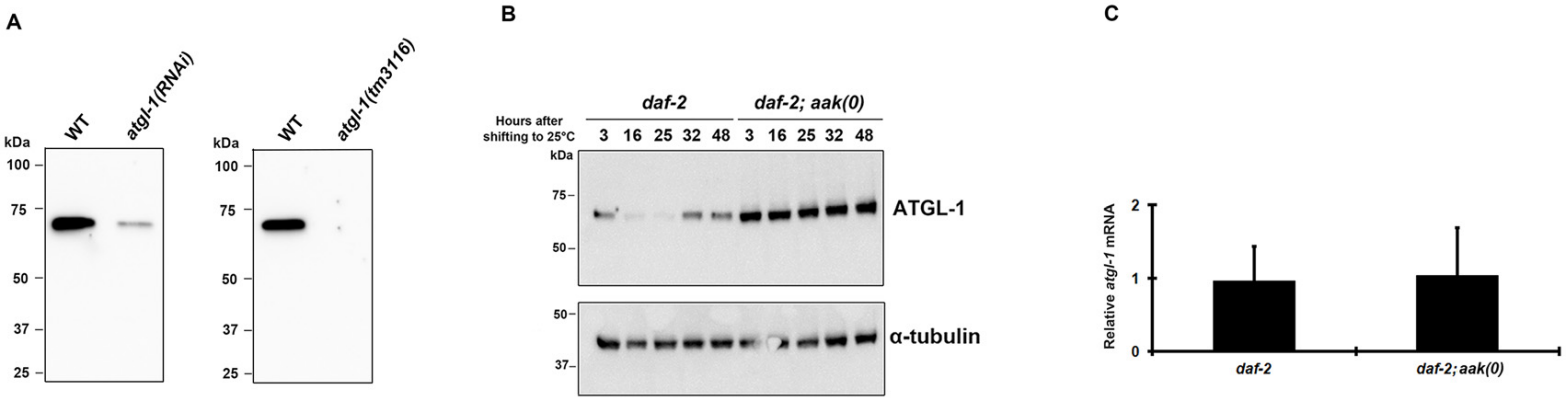
Endogenous ATGL-1 Protein is More Abundant in AMPK-deficient Dauer Larvae. **(A)** The ATGL-1 antisera recognizes a single band that migrates at approximately 70kD, which corresponds to the molecular weight of ATGL-1, and is eliminated in *atgl-1(RNAi)* animals. **(B)** Western blot analysis with *C*. *elegans* specific ATGL-1 antibody in *daf-2; aak(0)* mutant dauer larvae and control *daf-2* dauer larvae during the period of dauer entry. **(C)** mRNA Expression levels of ATGL-1 in control *daf-2* and *daf-2; aak(0)* dauers. Relative mRNA levels were analyzed by quantitative real-time PCR in dauer day 0 animals.

Using a polyclonal antibody raised specifically against *C*. *elegans* ATGL-1 **(** Fig 3A) we performed a Western blot analysis on day 0 control *daf-2* and *daf-2; aak(0)* dauer larvae. We noted that the endogenous ATGL-1 protein was significantly more abundant in the absence of AMPK at all the time points tested during the entire dauer entry period, consistent with the GFP expression analysis (Fig 3B). It is worth mentioning that the *atgl-1* mRNA levels were identical in both genetic backgrounds indicating that these differences resulted exclusively from post-transcriptional effects (Fig 3C).

**Fig 4:**
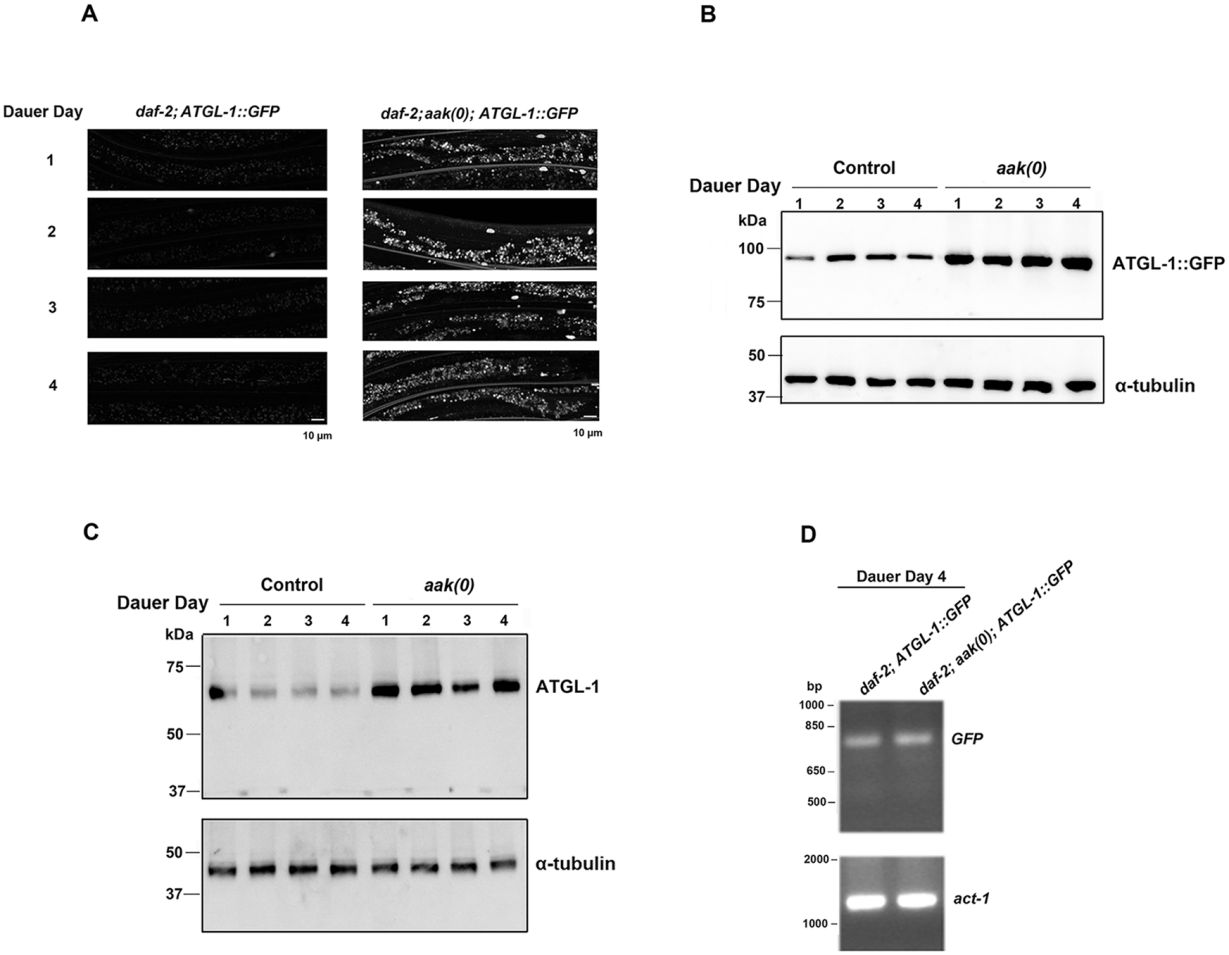
ATGL-1::GFP Expressed at a Higher Level in AMPK-deficient Animals during the Early Dauer Stage. **(A)** Comparison of ATGL-1::GFP levels between control *daf-2* and *daf-2; aak(0)* animals during the early dauer stage. Dauer day 1 is defined as 72 hours after shifting to restrictive temperature (25°C) at the L1 stage (see materials and methods). Scale bar = 10μm. **(B)** Western blot analysis of GFP levels in control *daf-2* and *daf-2; aak(0)* mutant animals during early dauer stage. **(C)** Western blot analysis of endogenous ATGL-1 levels in control *daf-2* and *daf-2; aak(0)* mutant animals during early dauer stage. **(D)** Quantification of ATGL-1::GFP mRNA levels in control *daf-2* and *daf-2; aak(0)* mutant dauer day 4 animals using semi-quantitative RT-PCR. *act-1* was used as loading control.

We also documented the ATGL-1::GFP levels (Fig 4A and 4B) and the endogenous ATGL-1 levels (Fig 4C) in the same animals during the early dauer stage from dauer day 1 (72 hours after shifting to restrictive temperature since L1 stage) to 4 and found that, similar to the dauer entry period, both levels were always more abundant in the larvae that lacked functional AMPK. Furthermore, the mRNA level of ATGL-1::GFP was also unchanged in control *daf-2* and *daf-2; aak(0)* dauer day 4 animals (Fig 4D). Therefore ATGL-1 levels are unlikely to be subject to feedback regulation and most probably increase in the absence of AMPK through post-transcriptional mechanisms. Taken together these data suggest that ATGL-1 is phosphorylated by AMPK, and this post-translational modification affects the enzyme by decreasing its stability.

### ATGL-1 Levels are Regulated in AMPK-mediated Phosphorylation and Subsequent Proteasome-Mediated Degradation

The AMPK-dependent change in ATGL-1 abundance suggests that the phosphorylation precedes elimination of the enzyme. Phosphorylation has been demonstrated to be an efficient means of targeting proteins for degradation therefore we tested whether AMPK-dependent phosphorylation targets ATGL-1 for proteasome degradation. To determine if the proteasome was involved in the observed elimination of ATGL-1 in the presence of AMPK we eliminated individual proteasome components by feeding RNAi and then used our anti-ATGL-1antibody to compare the endogenous ATGL-1 protein levels in control *daf-2* (AMPK-competent) and *daf-2; aak(0)* (AMPK-deficient) dauer larvae.

The proteasome contains one core protein-degrading 20S protein subunit comprising two proteasome alpha subunit (*pas*) rings and two proteasome beta subunit (*pbs*) rings; two 19S ATPase-like regulatory particles (*rpt*) responsible for stimulating the protein degradation activty of the 20S subunit by clearing the substrate entrance [20]; and one 11S non-ATPase-like regulatory particle (*rpn*) mainly required for peptide degradation. All these components contribute to optimal proteasome function.

**Fig 5:**
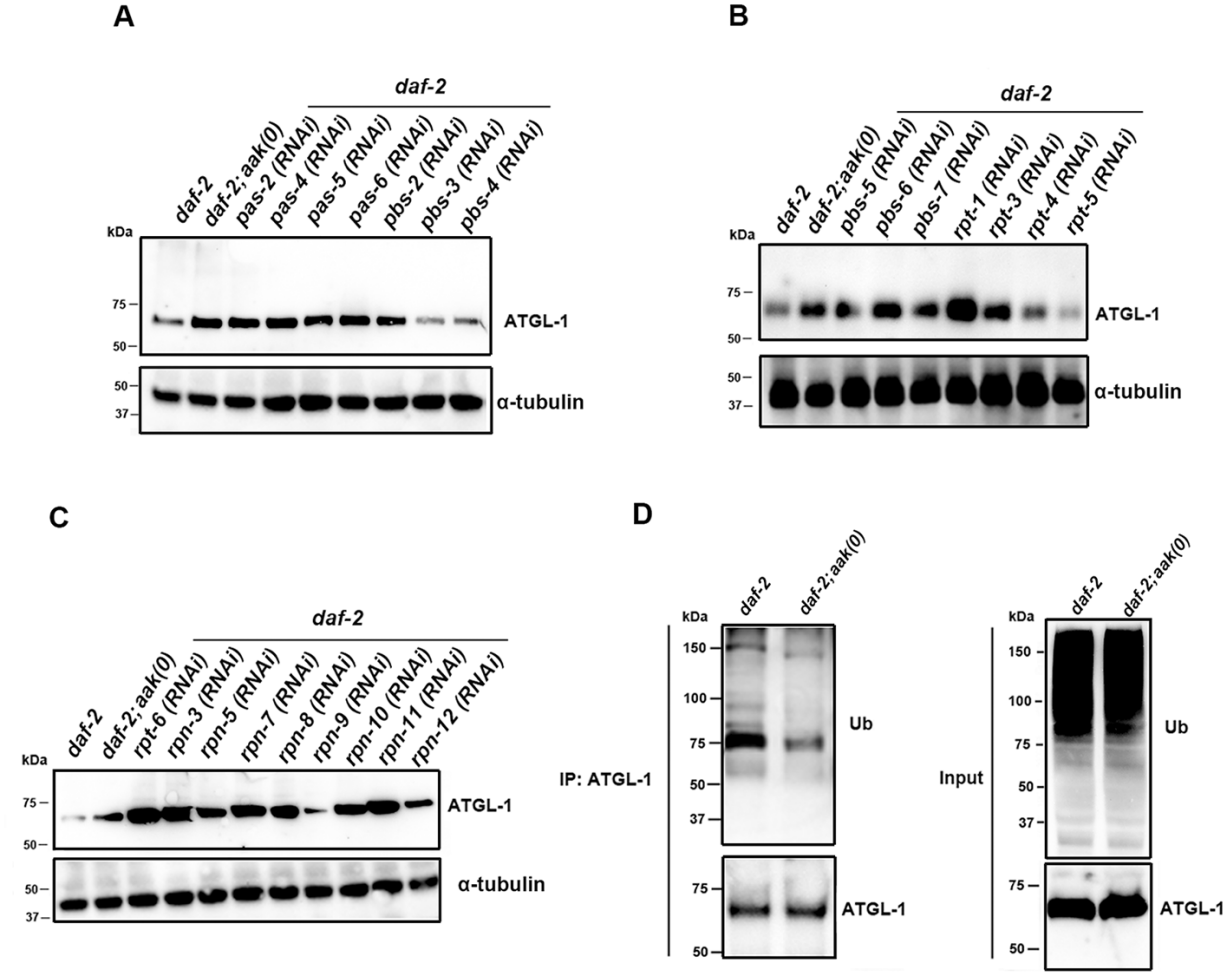
AMPK-Mediated Phosphorylation of ATGL-1 Enhances Ubiquitin-Mediated Degradation via the Proteasome. **(A)-(C)** ATGL-1 protein levels are regulated by AMPK by enhancing ubiquitin-mediated proteasomal degradation. Individual proteasome components were compromised through RNAi, of which many, but not all, increase the levels of ATGL-1 in control *daf-2* dauer larvae. **(D)** Ubiquitylated ATGL-1 intermediates accumulate in *daf-2; aak(0)* mutant dauer larvae. Immunoprecipitation of ATGL-1 from lysates obtained from both control *daf-2* and *daf-2; aak(0)* mutant dauer larvae was analyzed by western blot analysis using anti-ubiquitin antibody. High molecular weight ubiquitin-conjugated entities are seen in immunoprecipitates obtained from *daf-2; aak(0)* but are more prominent in the *daf-2* lysates. More lysate was loaded for control *daf-2* animals to equalize the amount of ATGL-1 protein loaded with that of *daf-2; aak(0)* animals. “IP:ATGL-1” refers to the protein lysate that was subjected to immunoprecipitation with our anti-ATGL-1 polyclonal antibody. “Input” refers to the total protein lysate before performing the immunoprecipitation step.

In separate RNAi experiments we compromised individual members of the *pas*, *pbs*, *rpt* and *rpn* gene families and noted that the elimination of most, but not all of these individual proteasome components were associated with increases in the levels of ATGL-1 protein in control *daf-2* dauers, whereby the levels became similar to those observed in *daf-2; aak(0)* dauers likely due to differential involvement of the various components [21], or potentially due to RNAi efficiency (Fig 5A–5C). This suggests that the AMPK-mediated reduction in ATGL-1 protein levels that we observed in control *daf-2* dauers requires a functional proteasome.

Given that many proteins are polyubiquitylated prior to proteasome-mediated degradation we next questioned whether this might also be the case for ATGL-1. We therefore immunoprecipitated ATGL-1 protein from whole *C*. *elegans* lysates obtained from control *daf-2* and *daf-2; aak(0)* dauer larvae and analyzed the precipitates by Western analysis using an ubiquitin-specific antibody. More protein was loaded for control *daf-2* animals to obtain an equal amount of ATGL-1 compared to *daf-2; aak(0)* animals. When normalized for the levels of ATGL-1 protein in the immunoprecipitates, we detected more ubiquitin associated with ATGL-1 in control *daf-2* dauer larvae compared to *daf-2; aak(0)* dauers, indicating that ATGL-1 is likely ubiquitylated in an AMPK-dependent manner prior to its degradation via the proteasome (Fig 5D).

### AMPK-Mediated Phosphorylation Causes ATGL-1 to Dissociate From the Lipid Droplets during the Dauer Stage

**Fig 6:**
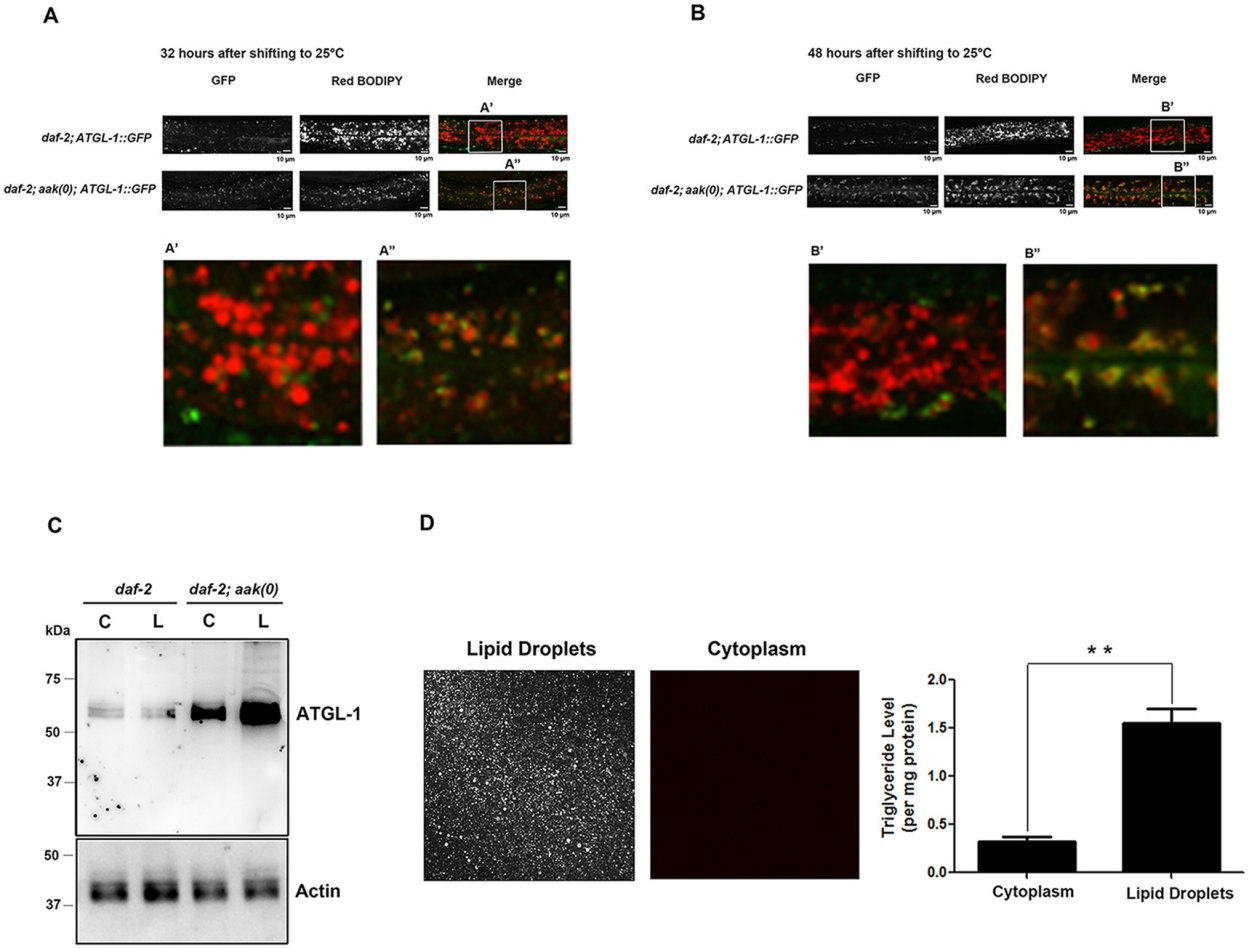
AMPK Regulates ATGL-1 Association with Lipid Droplets in Dauer Larvae. **(A)-(B)** Signal overlap between ATGL-1::GFP (Green) and C_1_-BODIPY-C_12_-stained lipid droplets (Red) was compared in control *daf-2* and *daf-2; aak(0)* mutant animals at 32 **(A)** and 48 hours **(B)** after shifting to restricted temperature. ATGL-1::GFP signal was closely associated with the labeled lipid droplets in *daf-2; aak(0)* mutant animals (white arrowheads in the insets) while the signals are clearly distinguishable from each other in control *daf-2* animals. Scale bar = 10μm. Insets were generated by selecting the same size of frame on each image and amplified by the same magnification. **(C)** Western blot analysis of the endogenous ATGL-1 levels in isolated lipid droplets (L) and cytoplasm (C) obtained from total day 0 (48 hours after shifting to restricted temperature) dauer extracts of control *daf-2* and *daf-2; aak(0)* mutant animals. Protein concentration was measured and 30μg of total protein was loaded in each sample lane. Actin was used as a loading control for the total protein level according to the recent proteomic study on *C*. *elegans* lipid droplets [39]. **(D)** Lipid droplet isolation method verified by significant C_1_-BODIPY-C_12_ staining and triglyceride enrichment in the isolated lipid droplets portion comparing to the cytoplasm (remaining portion of the total lysate) from *daf-2* day 0 dauer larvae.

Given that most triglyceride molecules are stored in the lipid droplets, we next determined whether ATGL-1 associates with the lipid droplets where it can initiate the lipolysis process, and whether this might be under AMPK-mediated regulation. We stained control *daf-2* and *daf-2; aak(0)* dauer larvae that expressed the ATGL-1::GFP translational fusion protein with red C_1_-BODIPY-C_12_ to label lipid droplets and subsequently monitored both fluorescent signals 32 and 48 hours after being shifted to restrictive temperature. In control *daf-2* dauer larvae, the ATGL-1::GFP signal was sequestered away from the red lipid droplet signal at both the 32 and 48 hour time points, whereas in *daf-2; aak(0)* dauers the ATGL-1::GFP signal still remained closely associated with the lipid droplets during the later part of the dauer entry period (Fig 6A and 6B).

To further confirm our observations, we isolated lipid droplets from intact animals of control *daf-2* and *daf-2; aak(0)* dauer day 0 larvae and compared the endogenous ATGL-1 protein levels in the isolated lipid droplet and the remaining cytoplasm fractions. The efficiency of the separation procedure and the quality of the isolated lipid droplets was verified by C_1_-BODIPY-C_12_ staining and triglyceride quantification of the cytoplasmic (C) and the lipid droplet fractions (LD) (Fig 6D). Following separation we observed that ATGL-1 protein was more abundant in the lipid droplet fraction of the *daf-2; aak(0)* dauers compared to the cytoplasmic fractions (Fig 6C), which provided a biochemical verification of our microscopic assessment. Little to no difference in ATGL-1 levels was observed in the lipid droplet and cytoplasm fractions of control *daf-2* dauers, likely due to the steady state low levels of ATGL-1 in these animals, potentially maintained through the continuous degradation of ATGL-1 (Fig 6C). Taken together, these results suggest that AMPK regulates the localization of ATGL-1 to limit its access to its triglyceride substrate in the lipid droplets.

### AMPK Phosphorylation Regulates 14-3-3 Protein Association with ATGL-1

**Fig 7:**
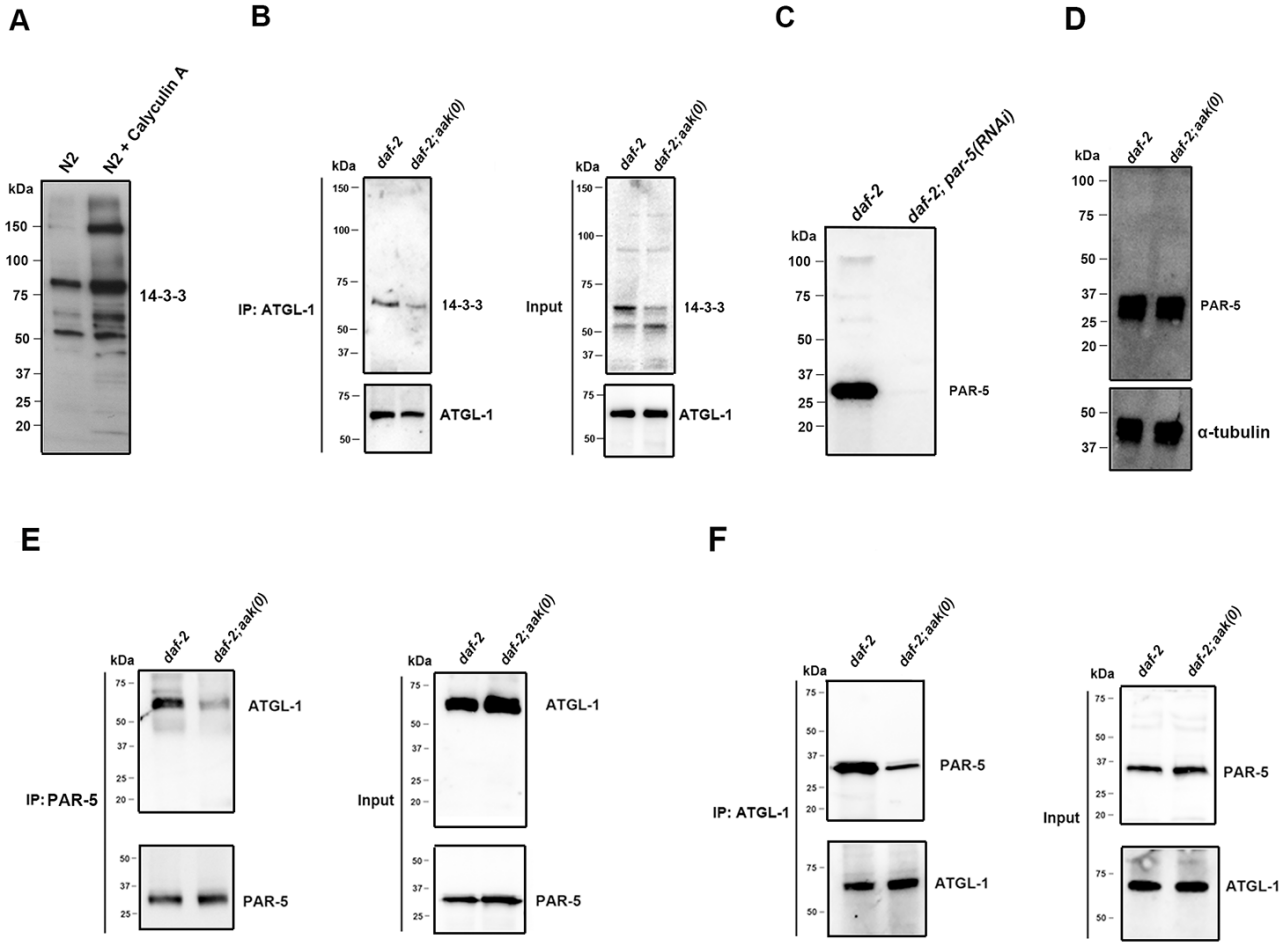
AMPK-Mediated Phosphorylation of ATGL-1 Generates More Phospho-(Ser) 14-3-3 Binding motifs and Enhances ATGL-1 Interaction with the *C*. *elegans* 14-3-3 Protein, PAR-5. **(A)** An increase in both the abundance and the number of bands that were detected by the P-14-3-3 antibody was observed in lysates obtained from N2 animals treated with the potent phosphatase inhibitor calyculin A. **(B)** More phospho-(Ser) 14-3-3 binding motif was generated in the ATGL-1 immunoprecipitates from control *daf-2* dauers compared to *daf-2; aak(0)* mutant dauers. Immunoprecipitation of ATGL-1 from total lysates obtained from control *daf-2* and *daf-2; aak(0)* mutant dauer larvae were immunoblotted with antisera that recognizes phospho-(Ser) 14-3-3 binding motif. More lysate was loaded for control *daf-2* animals to equalize the amount of ATGL-1 protein loaded with that of *daf-2; aak(0)* animals. “IP:ATGL-1” refers to the protein lysate that was subjected to immunoprecipitation with our anti-ATGL-1 polyclonal antibody. “Input” refers to the total protein lysate before performing the immunoprecipitation step. **(C)** The anti-PAR-5 antibody recognized a single band at approximately 30kD, corresponding to its predicted molecular weight, which disappeared following *par-5(RNAi)*. **(D)** PAR-5 protein levels are the same in control *daf-2* and *daf-2; aak(0)* dauers. **(E) and (F)** Immunoprecipitation of PAR-5 **(D)** or ATGL-1 **(E)** from protein lysates obtained from control *daf-2* and *daf-2; aak(0)* mutant dauer larvae were subjected to immunoblot analysis using ATGL-1 or PAR-5 antibody as indicated on the panels. More lysate was loaded for control *daf-2* animals to equalize the amount of ATGL-1 protein loaded with that of *daf-2; aak(0)* animals. “IP:ATGL-1” refers to the protein lysate that was subjected to immunoprecipitation with our anti-ATGL-1 polyclonal antibody. “Input” refers to the total protein lysate before performing the immunoprecipitation step.

Many of the downstream effects of AMPK phosphorylation that have been characterized are mediated through the generation of 14-3-3 protein binding sites followed by changes in subcellular localization [22, 23]. Therefore, since we observed a change in the localization of ATGL-1 in response to AMPK we questioned whether a similar mechanism might underlie the dissociation of ATGL-1 from the lipid droplets. We first performed bioinformatic analysis of the ATGL-1 protein sequence using the online Motif Scan tool (http://scansite.mit.edu/motifscan_seq.phtml), which revealed the presence of several regions that corresponded to potential 14-3-3 protein binding sites (data not shown). To address whether AMPK might generate 14-3-3 sites on ATGL-1 to dissociate it away from the lipid droplets, we analyzed our ATGL-1 immunoprecipitates from control *daf-2* and *daf-2; aak(0)* dauers with a 14-3-3 motif antibody that was generated against peptides bearing R-X-Y/F-X-pS sequence (Fig 7B). Western analysis suggested that 14-3-3 binding sites were less prominent in *daf-2; aak(0)* dauer larvae compared to control *daf-2* dauer larvae, consistent with a model wherein phosphorylation of ATGL-1 by AMPK generates a 14-3-3 site(s) that potentially leads to 14-3-3 Protein binding to modify ATGL-1 localization by dissociating it from the droplets (Fig 7B).

To directly verify the interaction between ATGL-1 and 14-3-3 protein, we performed immunoprecipitations on ATGL-1 and PAR-5, the major 14-3-3 protein homologue in *C*. *elegans* [24] (Fig 7C). Consistent with the observation that more 14-3-3 motifs are generated on ATGL-1 in control *daf-2* dauers, we found that more ATGL-1 was consistently immunoprecipitated by the PAR-5 specific antibody in control *daf-2* dauers compared to *daf-2; aak(0)* dauers (Fig 7E), while the reciprocal immunoprecipitation with our ATGL-1 antibody similarly yielded more PAR-5 in the precipitates in control *daf-2* dauer larvae (Fig 7F). Notably, total PAR-5 protein levels were unchanged in dauer larvae with or without functional AMPK (Fig 7D). Taken together, these data suggest that the AMPK-mediated phosphorylation generates 14-3-3 binding motifs on ATGL-1, which are subsequently recognized by 14-3-3/PAR-5 promoting association with ATGL-1 and subsequently triggering its dissociation from the lipid droplets.

### The Major Phosphorylation Site Ser303 on ATGL-1 Is Critical for Its Degradation and Subcellular Re-Localization

**Fig 8:**
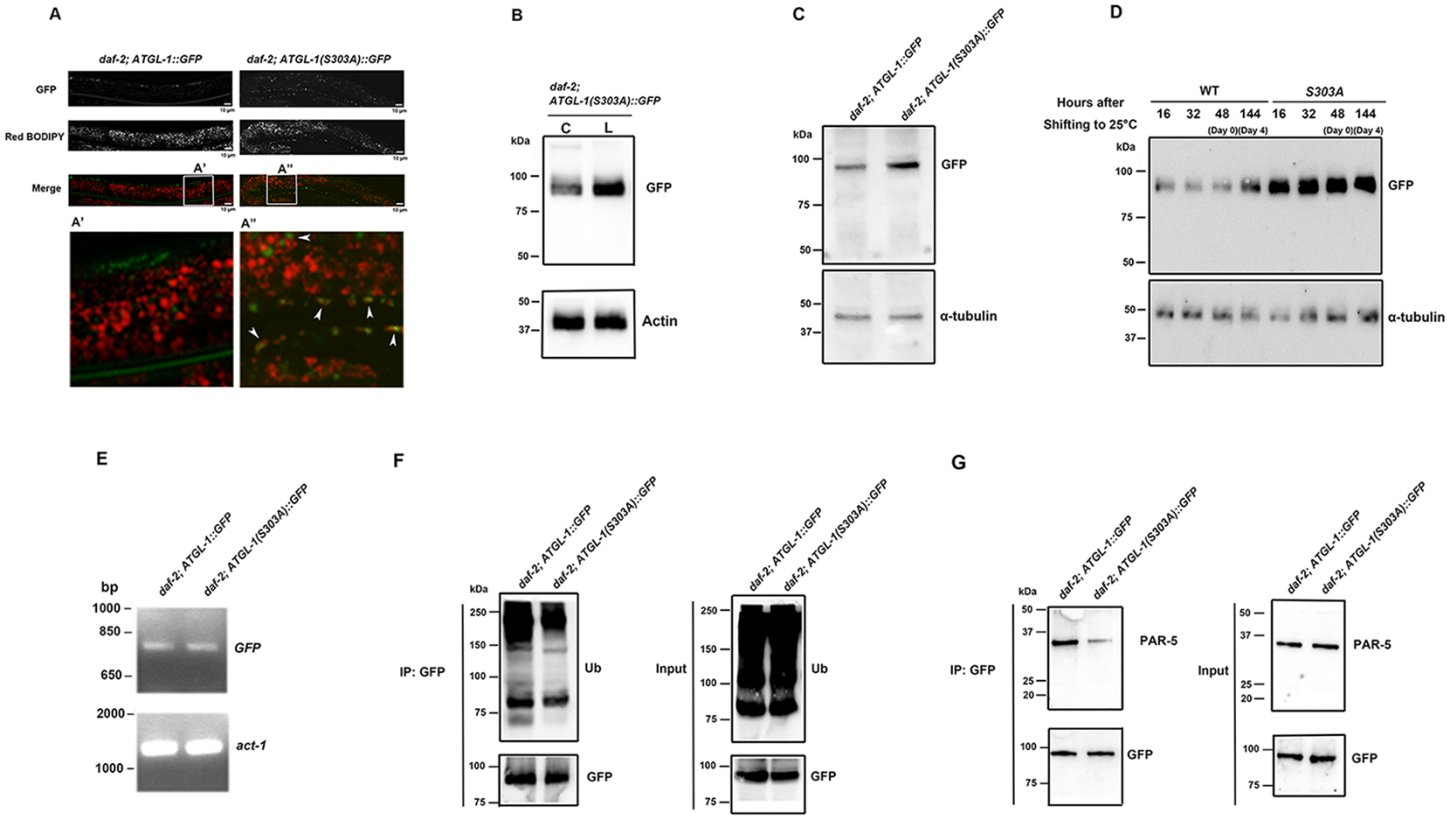
The Major AMPK Phosphorylable Site Ser303 on ATGL-1 Is Important for Its Intestinal Expression and Proteasome Degradation. **(A)** Expression patterns of ATGL-1::GFP and ATGL-1(S303A)::GFP were compared in control *daf-2* dauer larvae. Unlike ATGL-1::GFP, some ATGL-1(S303A)::GFP is expressed in hypodermis and intestine in S303A variants (white arrowheads in the insets). Scale bar = 10μm. Insets were generated by selecting the same size of frame and amplified to the same magnification. **(B)** Western blot analysis of GFP levels obtained from ATGL-1(S303A)::GFP in isolated lipid droplets (L) and cytoplasm (C) in control *daf-2* dauer day 0 larvae. **(C)** Western blot analysis of GFP levels obtained from ATGL-1::GFP and ATGL-1(S303A)::GFP in control *daf-2* dauer day 0 larvae. **(D)** Western blot analysis of GFP levels obtained from ATGL-1::GFP and ATGL-1(S303A)::GFP in control *daf-2* dauer larvae at different time points. **(E)** Quantiifcation of GFP mRNA levels in *daf-2; ATGL-1*::*GFP* and *daf-2; ATGL-1(S303A)*::*GFP* dauer day 0 larvae using semi-quantitative RT-PCR. *act-1* was used as loading control. **(F)** Immunoprecipitation of ATGL-1::GFP and ATGL-1(S303A)::GFP using GFP antibody from lysates obtained from *daf-2;* ATGL-1::GFP and *daf-2;* ATGL-1(S303A)::GFP dauer larvae was analyzed by western blot analysis using anti-ubiquitin antibody. “IP:GFP” refers to the protein lysate that was subjected to immunoprecipitation with an anti-GFP antibody. **(G)** Immunoprecipitation of ATGL-1::GFP and ATGL-1(S303A)::GFP using GFP antibody from protein lysates obtained from *daf-2;* ATGL-1::GFP and *daf-2;* ATGL-1(S303A)::GFP dauer larvae were subjected to Western analysis using PAR-5 antibody.

We previously demonstrated that ATGL-1 is directly phosphorylated by AMPK, at multiple sites of which Ser303 is the most critical for ATGL-1 activity [9]. Interestingly, bioinformatic analysis also revealed that the amino acid sequence at Ser303 corresponds to a strongly predicted 14-3-3 binding motif. To determine whether the degradation of ATGL-1 and its change of subcellular localization are directly linked to AMPK phosphorylation at the Ser303 site, we compared the subcellular localization and ATGL-1 levels in control *daf-2* dauer larvae expressing either wild type ATGL-1 or a non-phosphorylatable ATGL-1 variant (with Ser303 being mutated to Ala) tagged with GFP [9]. Unlike the control *daf-2* dauer expressing the WT ATGL-1::GFP, we found that some of the ATGL-1 variant GFP signal is still closely associated with lipid droplets especially within the hypodermis (Fig 8A). This association that we detected by fluorescence microscopy was further confirmed biochemically in the isolated lipid droplets where we detected an increased abundance of the ATGL-1 variant GFP signal in the lipid droplet fraction (L) compared to the cytoplasmic isolate (C) in control *daf-2* animals (Fig 8B). In addition, the level of GFP signal is much higher in the transgenic animals expressing the non-phosphorylatable ATGL-1 variant animals compared to those expressing the wild type ATGL-1 transgene (Fig 8C).

We also compared the GFP signal from the WT ATGL-1::GFP and the non-phosphorylatable ATGL-1::GFP variant at different time points during the dauer entry period and during the early dauer stage and observed higher GFP signal in the animals expressing the non-phosphorylatable ATGL-1 variant compared to the WT at all time points examined (Fig 8D). Unlike the protein levels, the mRNA levels of the two were unchanged in dauer day 0 animals (Fig 8E).

To compare the ubiquitylation levels on the WT ATGL-1::GFP and the ATGL-1 non-phosphorylatable variant, we immunoprecipitated ATGL-1::GFP or ATGL-1(S303A)::GFP protein from whole *C*. *elegans* lysates obtained from control *daf-2* dauer animals carrying the respective construct using a GFP antibody, and subjected the precipitates to Western analysis followed by blotting with a ubiquitin-specific antibody. When we normalized for the levels of WT or variant ATGL-1 protein in the immunoprecipitates, we detected more ubiquitin associated with WT ATGL-1 compared to the ATGL-1 variant (Fig 8F). Using the same precipitates blotted with the anti-PAR-5 antibody we also detected more PAR-5 protein associated with the WT ATGL-1 compared to the non-phosphorylatable variant (Fig 8G).

These results suggest that the major AMPK phosphorylation on ATGL-1 (site Ser303) is critical for the stability of ATGL-1. Because ATGL-1 remains associated with lipid droplets and is more abundant in *daf-2; aak(0)* dauer larvae, we suggest that the phosphorylation and the consequent increase in 14-3-3 binding are linked to the subsequent change in localization and eventually the degradation of ATGL-1, thus providing a switch-like mechanism that would protect the triglyceride stores for long term use during dauer.

## Discussion

Like many other organisms, *C*. *elegans* can overcome a multitude of environmental stresses during larval development by altering its developmental course to execute a motionless and non-feeding dauer stage accompanied by global developmental arrest. Once the dauer entry decision is made, the time the animals spend in the second larval stage (L2) is doubled to permit the animal to slow down its developmental and metabolic rate and to build up energy stores to prepare for the long-term period of nutrient deprivation [6]. In addition to these other processes the animals also undergo a progressive decrease in the rate of germline stem cell proliferation until the cells completely arrest allowing the animal to divert its energy resources normally devoted to reproduction, toward long-term survival. This trade off between reproductive ability and survival is tightly regulated by AMPK in the dauer larvae. Mutations that disrupt AMPK function give rise to a dramatic increase in germline stem cell proliferation and rapid consumption of the stored triglyceride energy stores, which eventually leads to the premature expiration of these mutant animals [8, 9].

*daf-2* dauers with compromised AMPK function demonstrate abnormally high ATGL-1 activity which accounts for the rapid exhaustion of the energy reserve and consequently premature expiration of the animals. AMPK plays an “energy protecting” role through phosphorylation of ATGL-1 in *C*. *elegans* dauer larvae, which we demonstrate has a two-fold effect: first, it generates 14-3-3 protein binding sites on ATGL-1 to sequester it away from its substrate, while the same phosphorylation also targets ATGL-1 for proteasome-mediated degradation.

AMPK-dependent proteasome-degradation has been previously documented in skeletal muscle and myocardial cells [17, 25], while the AMPK-dependent generation of 14-3-3 recognition sites has been well described during growth inhibition [22]. During the dauer stage AMPK uses these two mechanisms to safeguard the cellular energy reservoir from depletion by segregating the rate limiting lipolytic enzyme from its substrate and targeting it for degradation.

It is still unclear whether binding to PAR-5/14-3-3 is absolutely required for ATGL-1 ubiquitylation and degradation, however removal of ATGL-1 from the lipid droplets likely happens prior to its degradation. Notably, we observed a basal level of ubiquitylation and PAR-5/14-3-3 binding in the absence of AMPK, indicating that AMPK may not be the sole regulator of ATGL levels in dauer larvae. Recently, a newly identified lipid droplet protein called LID-1 was characterized as a regulator of lipolysis through its direct interaction with ATGL-1 [26]. LID-1 interacts with ATGL-1 to enhance its activity during periods of fasting outside the dauer stage. Although three gene products share significant sequence homology with CGI-58, LID-1 is the only one of the three that when compromised, it can block the reduction in lipid droplet volume and hence lipid hydrolysis during starvation, similar to loss of *atgl-1* function.

Previous work in human embryonic kidney cells demonstrated the generation of 14-3-3 binding sites on ATGL followed by interaction with a 14-3-3 protein downstream of AMPK phosphorylation in vitro [27]. However, 14-3-3 protein binding can modulate the function of a target protein in a number of different ways, including enzyme activity, subcellular localization, structure and stability, and molecular interactions [28]. Our data not only suggest a similar mechanism of ATGL-1 regulation in vivo to conserve the dauer-specific energy stockpile, but we go further to also reveal that this association precedes the change in subcellular localization and stability of the target ATGL-1 protein. Our finding reveals another means of regulating lipolysis in addition to the previously described involvement of the autophagy pathway, further highlighting the multifactorial regulation of lipolysis [29].

AMPK mutations are lethal in many other organisms examined to date, whereas AMPK-deficient *C*. *elegans* are viable and appear similar to wild type animals when they are not metabolically stressed, albeit they do have a modestly shortened life span [30]. However, its compromise becomes dramatically apparent during periods of energy stress like during dauer [9] and L1 starvation [31]. In these contexts the loss of AMPK renders the mutant animals more vulnerable to these challenges presumably due to their reduced capacity to adapt metabolically, thus leading to premature lethality, most likely due to lack of sufficient energy to drive major organ systems [9].

In mammalian adipocytes where most of our understanding of lipolysis has been derived, high energetic demands trigger activation of the β-adrenergic pathway to stimulate phosphorylation of both the lipid droplet-associated protein Perilipin1 and HSL by protein kinase A (PKA). This results in the translocation of HSL from the cytoplasm to the lipid droplets where it interacts with Perilipin1 and catalyzes triglyceride hydrolysis [32, 33]. Although ATGL activity is sensitive to β-adrenergic stimulation in adipocytes [34], unlike HSL it is not regulated by direct PKA phosphorylation [35], while the observed phosphorylation of Perilipin1 by PKA [36] may possibly involve translocation of ATGL to the lipid droplets [37].

In *C*. *elegans* AMPK-deficient dauer larvae we found that the loss of HSL had no effect on their survival, total lipid level, or lipid droplet size; all of which were modified in ATGL-1-deficient animals [9] (Fig 1H). This indicates that during periods of stress, when available energy levels are limited, lipolysis is tightly regulated, probably exclusively by ATGL-1, to initiate the first step of the process before the other lipase enzymes can exert their effects.

In mammals, one well-characterized role of AMPK in regulating cellular energy levels is by directly phosphorylating and inhibiting Acetyl-CoA Carboxylase (ACC) to terminate fatty acid synthesis [38]. Much of our current understanding of AMPK function in lipid homeostasis is rooted in its effects on fatty acid synthesis, while its role in hydrolysis is comparatively less well characterized.

AMPK activation often occurs in response to stresses that are commonly metabolic in nature, frequently arising from nutrient deprivation. Under these conditions it is most likely that limited or no nutrient/energy intake occurs in organism. Therefore, the inhibitory role of AMPK in fatty acid synthesis is probably secondary during these situations since the animals will simply not have the building blocks to synthesize and store fatty acids.

Although at first glance our data appear to contradict the role AMPK in regulating lipid homeostasis, we would argue that AMPK acts as a protective enzyme that may phosphorylate and modify pathways that may seem inconsistent with known functions of the enzyme. However, it is possible that because of its metabolic protective role these substrates and functions may indeed be developmental and/or physiological context-dependent; AMPK switches its role from promoting catabolism to preventing catabolism in specific contexts, for example by inhibiting ATGL-1 as demonstrated in *C*. *elegans* dauer.

Taken together, our study provides a detailed mechanistic account of how ATGL-1 is regulated by AMPK during periods of nutrient/energy deprivation and may shed light on how certain key enzymes that are involved in organismal energy management are regulated to fine-tune energy release according to physiological need.
